# Native Aortic Valve Infective Endocarditis Secondary to Community-Acquired Methicillin-Resistant Staphylococcus aureus: A Case Report and Literature Review

**DOI:** 10.7759/cureus.55341

**Published:** 2024-03-01

**Authors:** Saif Awlad Thani, Shahd M Al Jamei, Kadhiya N Al Azri, Khalid Al Alawi, Saud Al Shabibi

**Affiliations:** 1 Pediatric Critical Care, The Royal Hospital, Muscat, OMN; 2 Pediatrics, Oman Medical Specialty Board, Muscat, OMN; 3 Pediatric Cardiology, The Royal Hospital, Muscat, OMN; 4 Radiology, The Royal Hospital, Muscat, OMN

**Keywords:** methicillin-resistant staphylococcus aureus (mrsa), septic emboli, pediatric, aortic valve insufficiency, infective endocarditis

## Abstract

Infective endocarditis (IE) refers to a microbial infection affecting either a heart valve or endocardium, resulting in tissue damage and the formation of vegetation. Native aortic valve endocarditis in children is rare and is associated with serious complications related to valvular insufficiency and systemic embolizations. As reports about community-acquired methicillin-resistant *Staphylococcus aureus* (MRSA) native aortic valve endocarditis in children are very scarce, we report this case along with a literature review about its complications and management. Here, we report the case of a seven-month-old infant who was previously healthy and presented with signs and symptoms of shock and systemic embolizations secondary to native aortic valve IE. His blood culture showed MRSA. He developed aortic valve insufficiency heart failure and multiorgan septic emboli that progressed to fatal refractory multiorgan failure. The management of complicated aortic valve endocarditis in children is challenging and needs a multidisciplinary team approach and prompt intervention.

## Introduction

Infective endocarditis (IE) refers to a microbial infection affecting either a heart valve, natural or prosthetic, or the endocardium lining the heart’s inner walls, resulting in tissue damage and the formation of vegetation [1,2]. These vegetations consist of fibrin and clusters of bacteria and are responsible for damage to the heart structure and the potential formation of emboli [1,2]. The precise occurrence of IE in healthy children without any structural heart issues or predisposing factors remains uncertain. The reported proportion of IE in native valves out of total IEs has been reported to range from 19% to 33% [3,4]. Complications associated with IE include intracardiac, such as pseudoaneurysm, and extracardiac, such as central nervous system (CNS) embolization, leading to stroke or CNS infection [3-5]. Despite accessible guidelines for managing IE and improved handling of related problems, the mortality rate continues to be high, reaching up to 30%, with a higher risk of mortality in cases with neurological complications [3-5]. Native aortic valve endocarditis is rare in children, with few reported cases in children of methicillin-resistant *Staphylococcus aureus* (MRSA) aortic valve endocarditis [6,7]. The decision about surgery and the timing of surgery remains controversial and challenging, especially in patients with CNS complications [8,9]. Here, we report a rare case of a seven-month-old previously healthy infant with native aortic valve endocarditis secondary to community-acquired MRSA who developed severe aortic valve insufficiency and multiple fatal septic emboli. We performed a literature review of aortic valve endocarditis and its challenging management.

## Case presentation

A seven-month-old previously healthy boy with no prematurity, cardiac history, recurrent infections, hospital admissions, or any family history of immunodeficiency was brought to the emergency room (ER) with a four-day history of fever, decreased activity, and skin discoloration on the face, ear lobes, hands, and feet, which had progressed within a few hours before his presentation to the ER.

**Figure 1:**
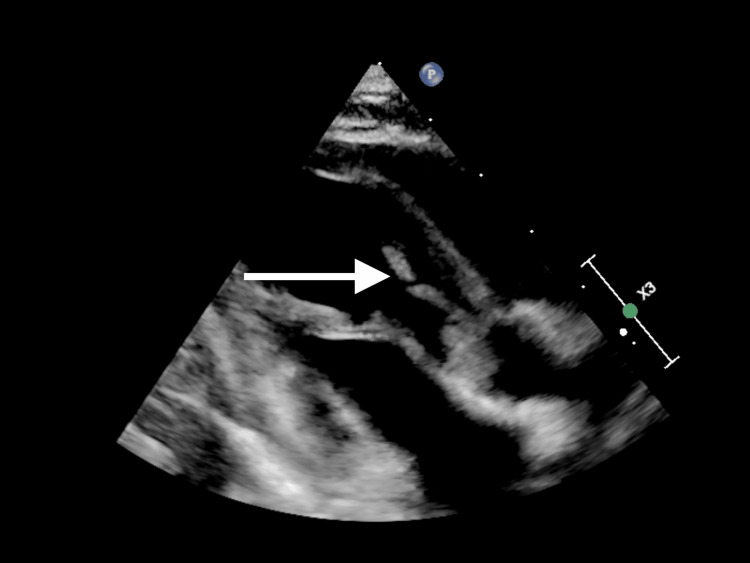
Parasternal long-axis two-dimensional echocardiographic view demonstrates a sizeable vegetation on the aortic valve extending into the left ventricle outflow tract.

**Figure 2:**
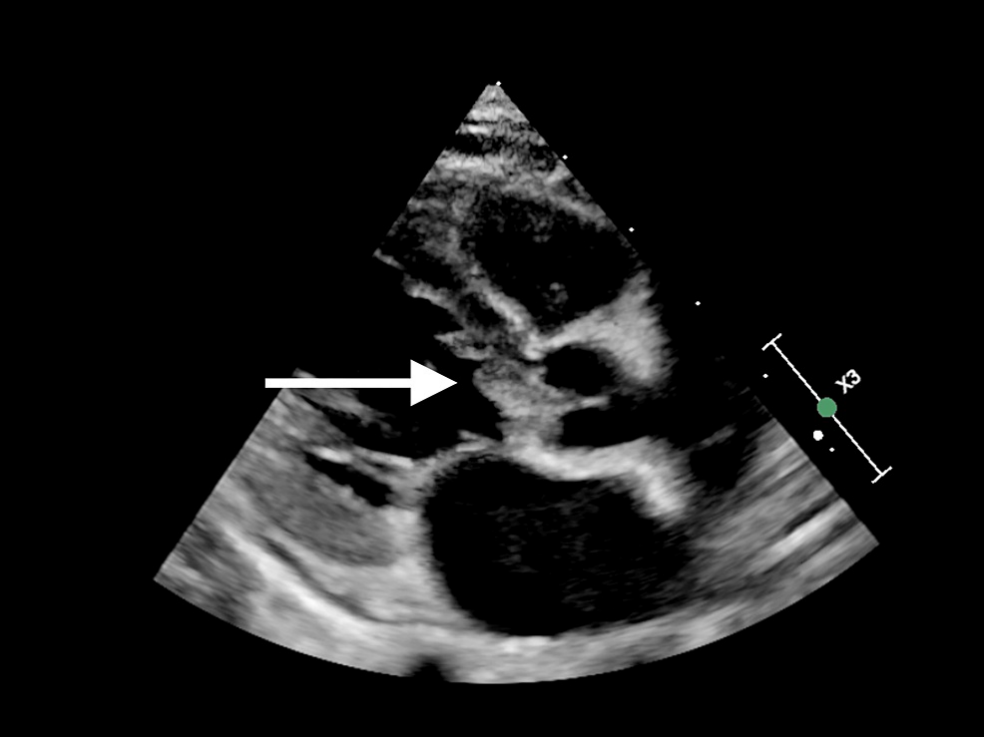
Another image demonstrating the vegetation on the aortic valve.

**Figure 3:**
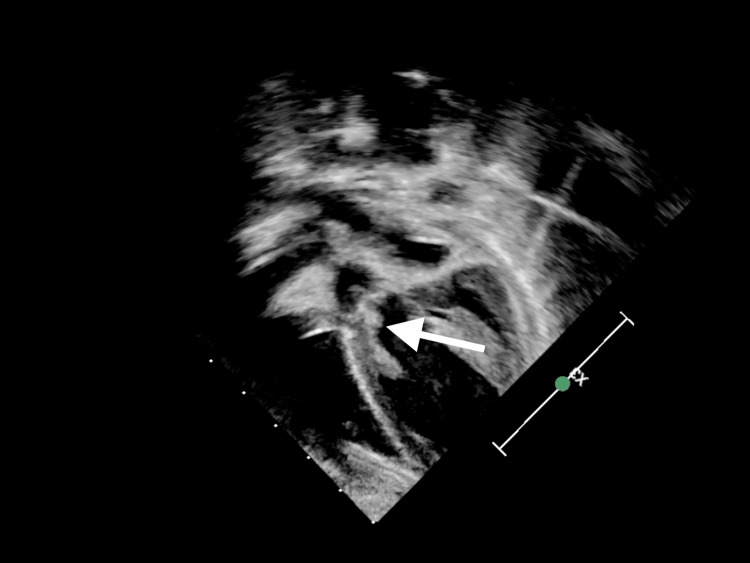
Modified five-chamber two-dimensional view showing the same vegetation.

**Figure 4:**
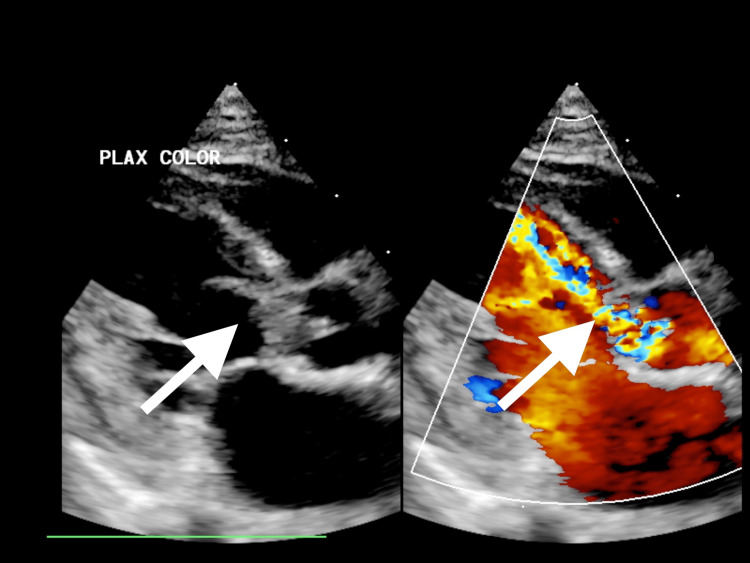
Long-axis parasternal view with color flow Doppler showing the same vegetation with significant aortic valve regurgitation due to destroyed aortic valve leaflets.

**Figure 5:**
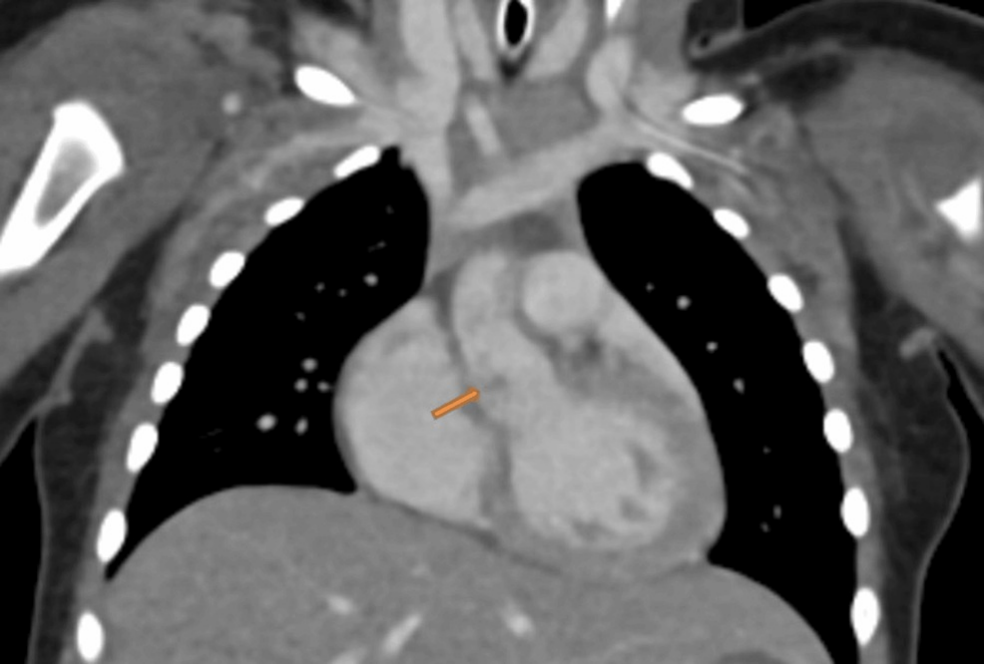
Contrast-enhanced thoracic computed tomography angiography shows evidence of aortic root dilatation with the presence of an ill-defined hypodense filling defect (arrow, presumed vegetation).

**Figure 6:**
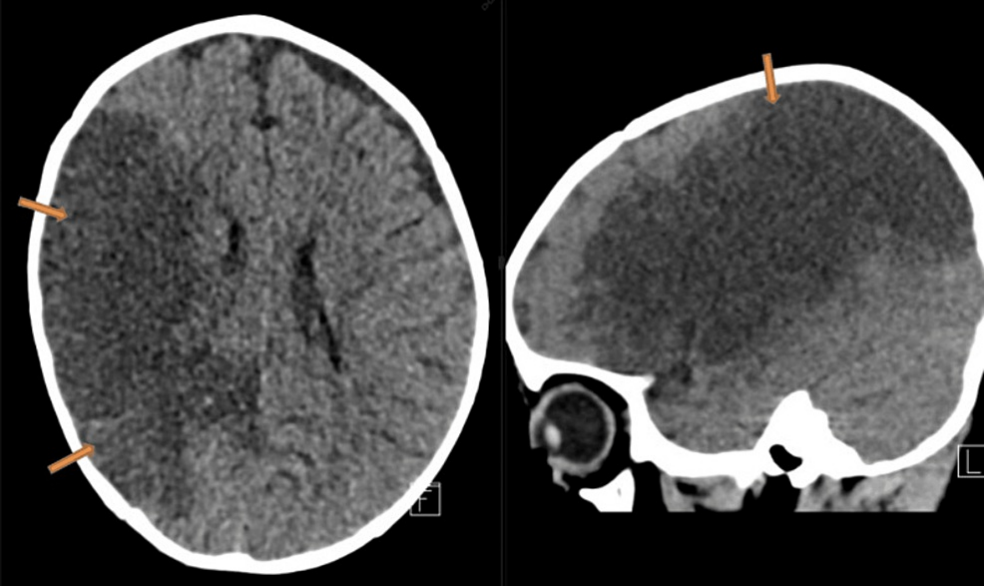
Selected axial image and sagittal multiplanar reconstructed image of a non-enhanced computed tomography of the brain shows evidence of a large area of hypoattenuation in the expected area of the right middle cerebral artery territory resulting in loss of gray-white matter differentiation.

Upon assessment, the patient exhibited signs of compensated shock with a purpuric rash on his legs, arms, and cheeks, accompanied by bluish discoloration on the tip of the nose, ear lobes, toes, and fingers. His Glasgow Coma Scale score was 10, and his pupils were asymmetric (1 mm on the left, 3 mm on the right). He was resuscitated with crystalloid and was given ceftriaxone and vancomycin. Initial blood gases showed metabolic acidosis with hyperlactatemia. Despite fluid boluses, his perfusion did not improve, and his lactate level continued to rise alongside persistent tachycardia. Consequently, he was started on adrenaline infusion, milrinone, and hydrocortisone. He was intubated and ventilated for depressed mental status and shock. Chest X-ray was normal. He had high C-reactive protein, anemia, thrombocytopenia, leucocytosis, transaminitis, high urea, and creatinine (Table 1). Within 21 hours, the blood culture showed MRSA. Echocardiography showed IE with huge vegetation in the aortic valve with severe aortic regurgitation and significant aortic root wall thickening. His aortic valve was found to be functionally bicuspid (Figures 1-4). Computed tomography (CT) of the chest showed presumed aortic root vegetation with ill-defined hypodensity measuring 13 mm × 9 mm with associated aortic root dilatation measuring around 15 mm compared to the normal size of 10 mm, with no other cardiac abnormality being identified (Figure 5). CT of the head showed right middle cerebral artery (MCA) territory acute infarction (Figure 6).

**Table 1:** Blood investigations upon admission.

Blood tests	Values	Reference range
Hemoglobin	6.3 g/dL	10.5–13.5 g/dL
Platelet	23 × 10^9^/L	140–400 × 10^9^/L
White blood cells	18.3 × 10^9^/L	6–17.5 × 10^9^/L
Neutrophils	8.7 × 10^9^/L	1.5–8.5 × 10^9^/L
Lymphocytes	6.4 × 10^9^/L	4–10.5 × 10^9^/L
C-reactive protein	285 mg/L	<10 mg/L
Urea	12 mg/L	1.8–6 mg/L
Creatinine	76 µmol/L	30–55 µmol/L
Alanine aminotransaminase	91 IU/L	10–40 IU/L
Aspartate aminotransferase	105 IU/L	34 IU/L
International normalized ratio	0.98	<1.5

**Figure 7:**
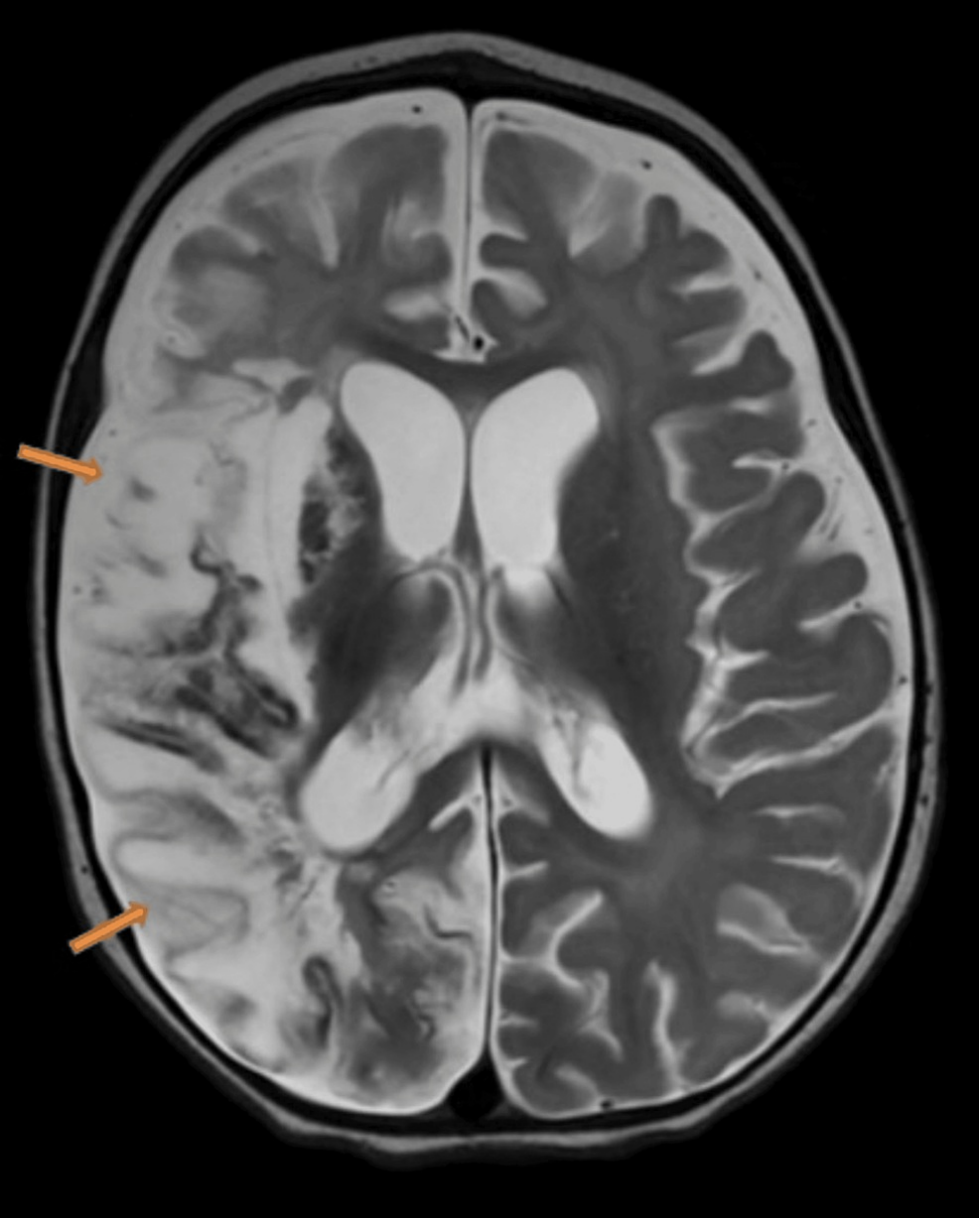
Axial transverse relaxation time (T2)-weighted image shows the expected evolution and sequel of the known previous right middle cerebral artery territory infarction, manifesting as encephalomalacia and gliosis.

**Figure 8:**
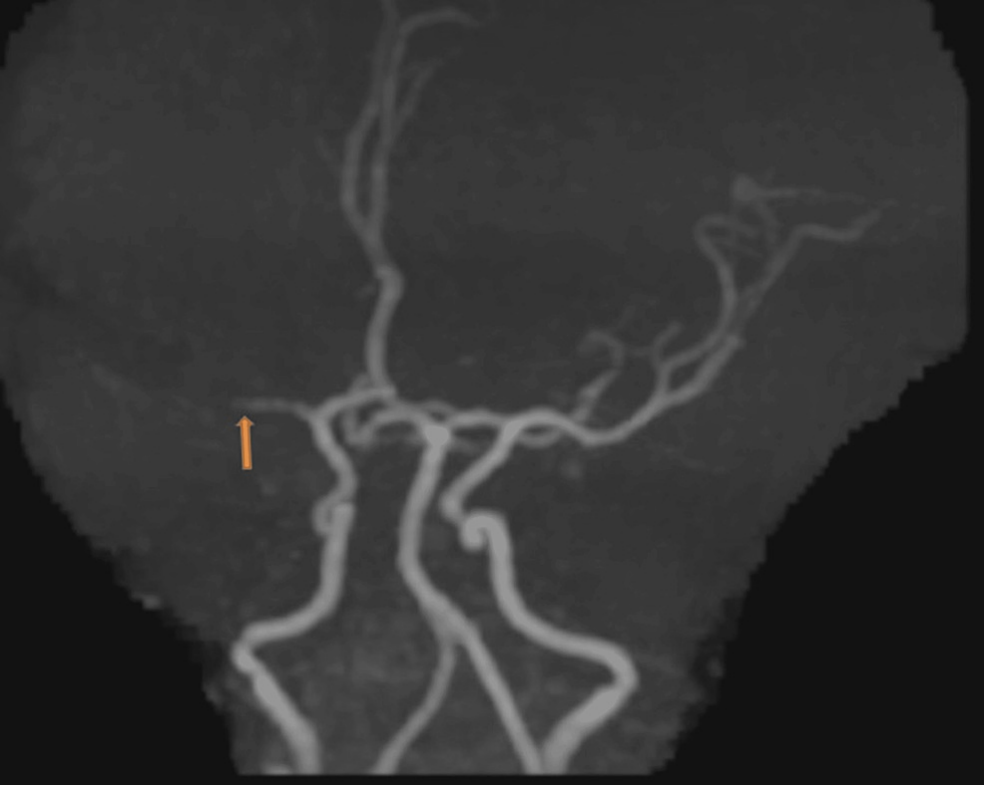
Time-of-flight magnetic resonance angiography shows a complete block of flow in the right middle cerebral artery.

**Figure 9:**
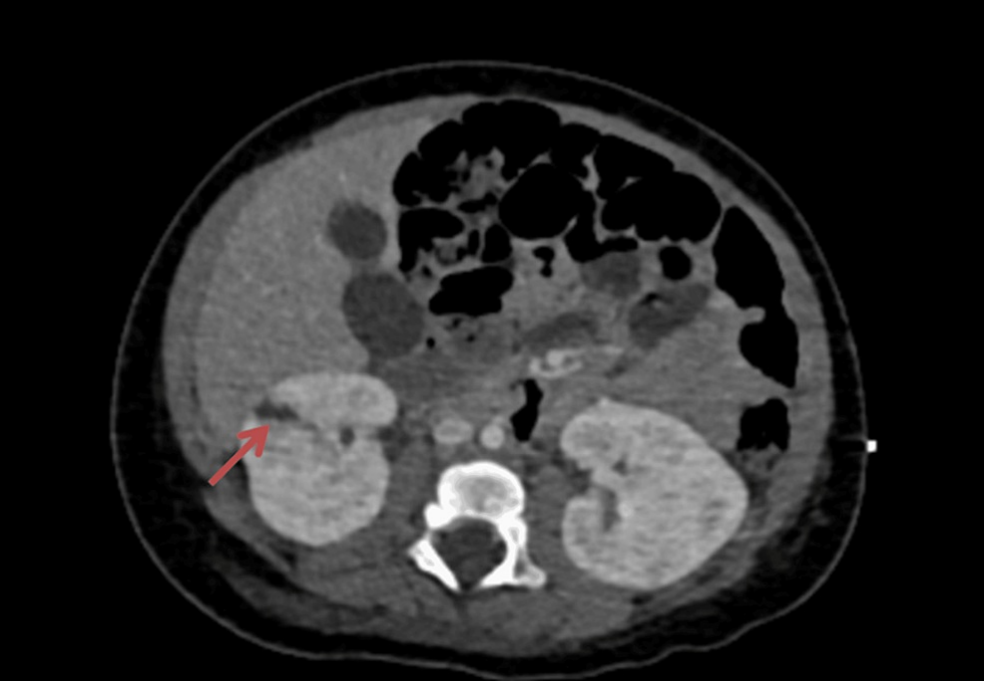
Contrast-enhanced computed tomography of the abdomen shows a small peripheral right renal wedge-shaped infarction (arrow).

The infectious diseases team was involved and changed the antibiotic to linezolid and rifampicin and added daptomycin. The blood culture was sterile after 10 days of admission. The patient was investigated for immunodeficiency disorders that resulted in normal tests. A cardiothoracic surgeon was consulted for surgical intervention, but due to the high risk of neurological complications with the Ross procedure during supra-systemic heparinization for cardiopulmonary bypass, it was decided to delay it and to re-evaluate the patient within two to four weeks. After four weeks and around the re-discussion and re-evaluation for the Ross procedure, magnetic resonance imaging (MRI) of the brain showed a complete block of flow in the right MCA with evolution and sequel of right MCA territory infarction (Figures 7, 8). He could not be weaned from positive pressure ventilation due to persistent heart failure. Because of multiple complications and organ failure, the surgical intervention was deemed to be futile with a high risk of mortality. During 40 days of pediatric intensive care stay, he developed gangrenous toes and fingers and had more septic emboli to the liver and kidney (Figure 9). Eventually, he had a fatal septic and cardiogenic shock with multiorgan failure that was refractory to all medical management.

## Discussion

We report a rare case of native aortic valve IE with multiorgan fatal septic emboli. Our case is rare due to five reasons. First, IE occurred in a previously healthy infant with normal heart structure who did not suffer from any cardiac symptoms since birth. IE is uncommon in children [7,10,11], especially in those with no underlying congenital heart disease [4,12,13]. Second, our patient had native aortic valve endocarditis which is rare in a healthy pediatric patient [14,15]. There are few reported cases of native aortic valve endocarditis in children [6,11,16-18]. Only one patient was aged below one year whose culture grew *Candida tropicalis* [18], making our patient one of the youngest patients who was reported to have aortic valve vegetation; this is the third distinctive feature of this case. Fourth, he was previously healthy with no prior hospital admission but his blood culture grew MRSA which was community-acquired. In a cohort of patients with IE without underlying heart disease, no patients had left-sided IE caused by MRSA [4]. As per our literature search, there was only one reported case in a nine-year-old patient with MRSA aortic valve endocarditis with a bicuspid valve [6]. Fifth, our patient had septic emboli to multiple organs (brain, liver, kidney, musculoskeletal), in addition to severe aortic valve insufficiency and heart failure, which made his management very challenging, especially the decision of aortic valve surgery.

Around 50-70% of IE in children is seen in those with congenital heart disease [19-21]. Patients at higher risk are those with cyanotic and complex congenital heart disease, left-sided defects, and endocardial cushion defects [22-25]. Although IE in children without underlying heart disease is rare [4], the use of central lines and other invasive procedures in neonatal and pediatric intensive care settings has led to an increased number of IE cases in children without underlying cardiac defects [2,26]. The triggering factors for IE in children include indwelling catheters, prematurity, cardiac structural defects, recent cardiac surgery, immunodeficiency, and malignancy [17,21,27,28]; however, our patient did not have any of these factors. Although MRSA has been known historically as a nosocomial infection, the epidemiology has changed, and it is no longer limited to hospitalized patients or persons with predisposing risk factors [29]. MRSA IE is uncommon and is described primarily among injection drug users with right-sided valvular lesions and in those with prosthetic valves [30-32]. MRSA native aortic valve endocarditis is rare and MRSA IE is reported predominantly in mitral valve [12].

IE can lead to many complications, and the prognosis depends on the type of causative organisms, the location and size of the vegetation, and emboli-related complications [2,4,7,33]. Worse prognosis and more complications are seen in previously healthy children compared to those with an identifiable predisposing condition which is probably related to a more aggressive pathogen [21]. Younger age with bacteremia is one of the predicting factors for a bad outcome. Our patient was only seven months old and his outcome was devastating. Aortic valve IE is uncommon in children, and the most predisposing lesion is a congenital bicuspid aortic valve and is associated with significant morbidity and mortality [1]. Aortic valve insufficiency and regurgitation, the severe complication that our patient suffered, results in heart failure which is more frequently encountered in in left-sided native valve IE [34-36]. Without operative intervention, heart failure secondary to valvular insufficiency is the leading cause of death due to progressive hemodynamic deterioration [1,20]. Aortic valve vegetation leads to systemic embolization and affects many organs, including the brain, liver, kidney, spleen, and musculoskeletal system. Most embolic events affect the CNS, mainly in the distribution of the MCA, which is associated with hospital mortality [37]. Vegetation size, location, mobile vegetation, age, and bacteremia are among the predictors of embolic events [38-40]. More systemic embolism was found in patients with non-congenital heart disease, and, in many instances, embolism is already present at the time of diagnosis [4,39,41]. Our patient had features of embolism on presentation. He had bluish discoloration on the tip of the nose, fingers, and toes and was found to have a stroke on the initial head CT.

Most children with IE are treated with antibiotics and supportive care only [42]. The decision about surgical intervention and the timing of surgery for IE is challenging, especially in the presence of CNS complications. The mortality rates may approach 50% [8,43,44]. The indications for surgical intervention include refractory heart failure, severe valvular insufficiency, recurrent septic embolism, large mobile vegetations, the presence of a prosthetic valve, and persistent sepsis despite adequate antibiotics [8,43-46]. Aortic valve surgery for children with IE may include valve repair, the Ross procedure, a homograft root replacement, or a mechanical valve replacement [10,15]. Case reports and studies have reported survival and good outcomes among patients who underwent surgical intervention for aortic valve endocarditis [1,5,10,15,17,47]. In our patient, the presence of stroke, the fear of hemorrhagic transformation during bypass surgery, and the high risk of mortality due to the presence of multiple systemic embolizations prevented surgical intervention. The timing of surgery for IE in the presence of stroke remains controversial, and some guidelines suggest delaying it for up to four weeks [9,48], which is not possible in case of severe heart failure with failed medical management or if the patient continues to have systemic embolization. Some studies have suggested early surgery in high-risk patients even in the presence of ischemic stroke, showing it is safe and not associated with neurological deterioration or worse outcomes [8,49,50]. Unfortunately, without surgery, our patient continued to deteriorate with severe heart failure secondary to aortic valve insufficiency and developed multiple septic emboli and multiple organ failures that led to death. Referral for early surgery and multidisciplinary team discussion and decision before progression to refractory heart failure and multiple septic emboli is paramount in these cases.

## Conclusions

Native aortic valve IE secondary to community-acquired MRSA is rare in previously healthy children. It is associated with heart failure secondary to valvular insufficiency and leads to septic emboli in multiple organs. Although the decision about operative intervention remains challenging, early intervention and a multidisciplinary approach before the progression to refractory heart failure and irreversible organ damage due to systemic embolization may improve patient outcomes.
